# Hepatitis B surface antigen levels: association with 5-year response to peginterferon alfa-2a in hepatitis B e-antigen-negative patients

**DOI:** 10.1007/s12072-012-9343-x

**Published:** 2012-03-23

**Authors:** Patrick Marcellin, Ferruccio Bonino, Cihan Yurdaydin, Stephanos Hadziyannis, Rami Moucari, Hans-Peter Kapprell, Vivien Rothe, Matei Popescu, Maurizia R. Brunetto

**Affiliations:** 1 Service d’Hépatologie and INSERM U773-CRB3, Hôpital Beaujon, University of Paris, 100 boulevard du Général Leclerc, 92110 Clichy, France; 2 Liver and Digestive Disease Division, Department of Internal Medicine, University Hospital of Pisa, Pisa, Italy; 3 Faculty of Medicine, University of Ankara, Ankara, Turkey; 4 Department of Medicine and Hepatology, Henry Dunant Hospital, Athens, Greece; 5 Abbott GmbH & Co, Wiesbaden-Delkenheim, Germany; 6 IST GmbH, Mannheim, Germany; 7 F Hoffmann-La Roche, Basel, Switzerland; 8 Hepatology Unit, University Hospital of Pisa, Pisa, Italy

**Keywords:** Chronic hepatitis B, HBsAg clearance, HBV DNA suppression, Long-term response, Prediction

## Abstract

**Purpose:**

To investigate the durability of response to peginterferon alfa-2a up to 5 years post-treatment and factors associated with response in hepatitis B e-antigen (HBeAg)-negative patients.

**Methods:**

HBeAg-negative patients received peginterferon alfa-2a (180 μg/week) ± lamivudine (100 mg/day) for 48 weeks as part of a multicenter, randomized study. The planned 5-year efficacy analysis included patients (*n* = 230) enrolled in the long-term follow-up study. On-treatment hepatitis B surface antigen (HBsAg) decline kinetics were analyzed retrospectively in a subgroup of patients with HBsAg data available at baseline, weeks 12, 24, and 48 on-treatment, and 6 months post-treatment (*n* = 120). Receiver operating characteristic analyses identified the on-treatment HBsAg levels associated with response at 1 and 5 years post-treatment.

**Results:**

HBV DNA ≤2,000 IU/mL and HBsAg clearance at 5 years post-treatment were achieved by 23 and 12% of patients, respectively. High rates of HBsAg clearance at 5 years post-treatment were achieved by patients with HBV DNA ≤2,000 IU/mL at 1 year post-treatment (28%). Rates of HBV DNA ≤2,000 IU/mL at 1 year post-treatment were 47.2 and 43.4% in patients with ≥10% decline from baseline at weeks 12 and 24, respectively, compared with 16.4% (*p* = 0.0003) and 13.2% (*p* < 0.0004) in patients with a <10% decline. Rates of HBsAg clearance at 5 years post-treatment were 22.6 and 22.4% in patients with ≥10% decline at weeks 12 and 24, respectively, compared with 7.5% (*p* = 0.0161) and 3.8% (*p* < 0.0001) in patients with <10% decline.

**Conclusions:**

Peginterferon alfa-2a results in increasing rates of HBsAg clearance during post-treatment follow-up in HBeAg-negative patients. On-treatment decline in HBsAg is significantly associated with long-term post-treatment response.

## Introduction

Chronic hepatitis B virus (HBV) infection is estimated to affect 350 million people worldwide and is associated with development of cirrhosis and hepatocellular carcinoma [1]. Serum hepatitis B surface antigen (HBsAg) is usually used as a qualitative marker for the diagnosis of HBV infection, and chronic hepatitis B (CHB) is defined as persistence of HBsAg in the circulation for more than 6 months. HBsAg clearance is considered a marker of complete and definitive remission of HBV activity [2], because it is associated with reduced incidence of hepatocellular carcinoma and improved rates of survival, and is the closest outcome to clinical cure of CHB [3–5].

Previous studies with conventional interferon alfa-2a in patients with hepatitis B e-antigen (HBeAg)-negative CHB demonstrated that HBsAg clearance could be achieved by this population. Approximately, one-third of patients who sustained a biochemical response for a median period of 7 years post-treatment achieved HBsAg clearance [6]. Subsequently, a finite course of peginterferon alfa-2a has been shown to result in HBsAg clearance, which increases in the years following the completion of the course of therapy. Indeed, rates of HBsAg clearance have been shown to reach 9% at 3 years post-treatment in HBeAg-negative patients [7]. Although HBsAg clearance is the ultimate goal in HBeAg-negative CHB [8], rates of response in the short-term are low and, therefore, it is not usually the primary efficacy endpoint in clinical studies. Alternative markers of response have been identified that are associated with subsequent increased rates of HBsAg clearance and long-term clinical benefits. In HBeAg-negative CHB, HBV DNA suppression ≤2,000 IU/mL is used to define sustained immune control with pegylated interferon in the shorter term (e.g., 1 year post-treatment) as this level of HBV DNA is associated with inactive disease and a low risk of hepatocellular carcinoma [2, 9, 10]. Patients who achieve sustained immune control following interferon-based therapy are likely to clear HBsAg during long-term treatment-free follow-up [11, 12].

The pivotal study of peginterferon alfa-2a in HBeAg-negative patients showed that approximately one-third of patients achieve sustained immune control [13]. Identification of responders either before or early during treatment would be of great benefit as it would not only ensure timely initiation of treatment in patients likely to respond but also allow modification of the treatment regimen in those patients unlikely to respond to the standard duration (48 weeks) of peginterferon alfa-2a monotherapy. Recent analyses have shown that serologic (e.g., HBsAg) and virologic (e.g., HBV DNA) markers either before or during treatment with pegylated interferon may help identify those patients most likely to respond post-treatment [14]. In a retrospective study, it was also shown that low pretreatment HBsAg levels and their decline during treatment with conventional interferon alfa predicted subsequent HBsAg clearance [15].

The current analysis describes the response rate at 5 years post-treatment in HBeAg-negative patients treated with peginterferon alfa-2a during the Phase 3 study—this is the final planned efficacy analysis from this pivotal trial. In addition, it investigates the potential of HBsAg quantification during treatment to predict long-term response.

## Materials and methods

### Study design

HBeAg-negative patients received peginterferon alfa-2a (180 μg/week) ± lamivudine (100 mg/day) for 48 weeks as part of the large, multicenter, randomized Phase 3 study [13]. Efficacy was assessed as part of the long-term follow-up study [7], and results at 5 years post-treatment are reported. Additional retrospective analyses of data from the initial and long-term study were also conducted.

HBsAg levels were analyzed retrospectively from stored samples collected at baseline, during therapy at weeks 12, 24, and 48 (end of treatment), and 6 months post-treatment (week 72). HBsAg levels were quantified using the Abbott Architect HBsAg assay (Abbott Laboratories, IL, USA; dynamic range 0.05–250.0 IU/mL) after 1:100 dilution. Samples with HBsAg >250.0 IU/mL at this dilution were retested at a final dilution of 1:1,000. Samples with HBsAg levels <0.05 IU/mL at 1:100 dilution were retested at the same dilution [16]. HBV DNA was measured using the AMPLICOR HBV test (Roche Molecular Diagnostics, Pleasanton, CA, USA; range 71–35,715 IU/mL); samples with HBV DNA >35,715 IU/mL were retested after 1:100 dilution according to the manufacturer’s instructions.

### Efficacy endpoints

The original protocol stated that post-treatment efficacy should be determined at yearly time-points with the final assessment taking place 5 years post-treatment. The efficacy endpoints included in the current analysis were HBV DNA ≤2,000 IU/mL (≈10,000 copies/mL)—a marker of sustained immune control—and HBsAg clearance at 1 and 5 years post-treatment. Both parameters were assessed in patients enrolled in the long-term follow-up study (*n* = 230), and in patients included in the long-term follow-up study with HBsAg values available at baseline, weeks 12, 24, and 48 of treatment and 6 months post-treatment (*n* = 120). The HBsAg kinetic analysis was conducted in the subgroup of 120 patients with HBsAg levels available at all time-points.

### Association of on-treatment HBsAg and HBV DNA levels with response post-treatment

As HBsAg decline from baseline has previously been shown to be similar in peginterferon alfa-2a and peginterferon alfa-2a + lamivudine-treated patients [17], data from both treatment groups were pooled. HBV DNA decline has been shown to be greater in patients treated with peginterferon alfa-2a + lamivudine compared with patients treated with peginterferon alfa-2a alone [17] and, therefore, wherever HBV DNA decline was assessed, the two treatment arms were analyzed individually.

Only patients with HBsAg values available at baseline and at all other time-points (weeks 12, 24, 48, and 72) were included in this analysis. The association between HBsAg decline during treatment and response at 1 and 5 years post-treatment was investigated. Association between HBsAg decline and response at 6 months post-treatment was not investigated, due to the high rate of relapse between 6 months and 1 year post-treatment [7].

### Statistical analysis

Receiver operating characteristic analysis at weeks 12 and 24 of therapy was used to identify levels of HBsAg decline from baseline associated with high rates of response post-treatment. The target was to identify a cut-off value that would provide a negative predictive value (NPV) ≥95%.

Logistic regression was used to analyze the chance of a response. In general, missing samples were treated as non-response. The only exception was when calculating the rate of HBsAg clearance, where last-observation-carried-forward (LOCF) methodology was used in patients with HBV DNA ≤71 IU/mL at the missing time-point.

All statistical tests were considered exploratory and no adjustment for multiple testing was performed. Mantel–Haenszel Chi-square (MHχ²) test and Wald Chi-square (WCχ²) test were used as appropriate. The statistical analysis software used was SAS version 8.0 (SAS Institute, Cary, NC, USA).

## Results

A total of 230 peginterferon alfa-2a ± lamivudine-treated patients were included in the long-term follow-up study. Of these, 120 (52%) had HBsAg values available at all time-points (i.e., baseline and weeks 12, 24, and 48 of therapy and 6 months post-treatment). Of the patients with HBsAg values available at all time-points, the majority (78%) was from the Asia–Pacific region. Baseline characteristics were similar between the two populations (Table 1).

**Table 1:** Baseline characteristics for peginterferon alfa-2a ± lamivudine-treated patients included in the long-term follow-up study (*n* = 230) and in those with HBsAg values at each time-point (*n* = 120)

	Long-term follow-up population^a^ (*n* = 230)	Long-term follow-up population with HBsAg available at all time-points^b^ (*n* = 120)
Ethnicity (%) Caucasian/Asian/other	27/72/1	32/67/2
Gender (%) Male/female	83/17	75/25
Genotype^c^ (%) A/B/C/D	7/28/42/20	10/20/46/22
Age (years) mean ± SD	39.9 ± 11.0	41.3 ± 9.9
HBsAg (log_10_ IU/mL) mean ± SD	3.39 ± 0.61	3.40 ± 0.61
HBV DNA (log_10_ IU/mL) mean ± SD	6.46 ± 1.91	6.49 ± 1.85
ALT (IU/L) mean ± SD	87 ± 75	92 ± 87
Number of patients with HBsAg values available at all time-points	NA	120
China		32
Hong Kong		26
Thailand		13
Italy		11
Poland		10
Spain		9
Turkey		7
Taiwan		5
France		4
New Zealand		2
Greece		1

*ALT* alanine aminotransferase, *NA* not applicable

^a^Ref. [7]

^b^Patients with HBsAg values at baseline and at weeks 12, 24, 48, and 72

^c^Seven patients in the overall population and three patients in the current analysis population were not infected with one of the four main HBV genotypes

### Efficacy of peginterferon alfa-2a at 1 and 5 years post-treatment

In the long-term population (*n* = 230), 72 patients (31%) had HBV DNA ≤2,000 IU/mL at year 1 post-treatment and 11 patients (5%) had cleared HBsAg (Table 2). In the 72 patients with HBV DNA ≤2,000 IU/mL at year 1 post-treatment, 50% (36/72) had sustained suppression of HBV DNA ≤2,000 IU/mL at year 5 post-treatment. For comparison, in patients with available data at year 1 and year 5 post-treatment, 88% (36/41) sustained suppression of HBV DNA ≤2,000 IU/mL. The rate of HBsAg clearance at 5 years post-treatment was significantly higher in patients with HBV DNA ≤2,000 IU/mL at 1 year post-treatment (20/72; 28%) than in patients with HBV DNA >2,000 IU/mL at 1 year post-treatment (8/158, *p* < 0.0001). HBsAg clearance was achieved by 42.4% (14/33) of patients with HBV DNA <70 IU/mL at 1 year post-treatment.

**Table 2:** Response rates at 1 and 5 years post-treatment for peginterferon alfa-2a ± lamivudine-treated patients included in the long-term follow-up study (*n* = 230) and for patients included in the current analysis (*n* = 120)

	Long-term follow-up population^a^ (*n* = 230)	Long-term follow-up population with HBsAg available at all time-points^b^ (*n* = 120)
Response at 1 year post-treatment, *n* (%)		
HBV DNA ≤2,000 IU/mL	72 (31)	36 (30)
HBsAg clearance	11 (5)	6 (5)
Response at 5 years post-treatment, *n* (%)		
HBV DNA ≤2,000 IU/mL	54 (23)	31 (26)
HBsAg clearance	28 (12)	17 (14)

^a^Ref. [7]

^b^Patients with HBsAg values at baseline and at weeks 12, 24, 48, and 72

In patients with HBsAg values available at all time-points (*n* = 120), 36 patients (30%) had HBV DNA ≤2,000 IU/mL at year 1 post-treatment and, of these, 14 (39%) achieved HBsAg clearance at 5 years post-treatment (compared with 3.6% (3/84) of patients with HBV DNA >2,000 IU/mL at 1 year post-treatment, *p* < 0.0001). Rate of HBsAg clearance in patients with HBV DNA <70 IU/mL at 1 year post-treatment was 61.5% (8/13). Rates of response at 1 and 5 years post-treatment in both populations are shown in Table 2.

### Baseline HBsAg as a predictor of response

Receiver operating characteristic analysis identified a baseline HBsAg level of 5,000 IU/mL that was associated with post-treatment response. This provided a positive predictive value (PPV) of 34% for HBV DNA ≤2,000 IU/mL at 1 year post-treatment and a PPV of 30% for HBV DNA ≤2,000 IU/mL at 5 years post-treatment. The NPVs generated were 78 and 84%, respectively.

### Association between HBsAg decline and post-treatment response

**Fig. 1:**
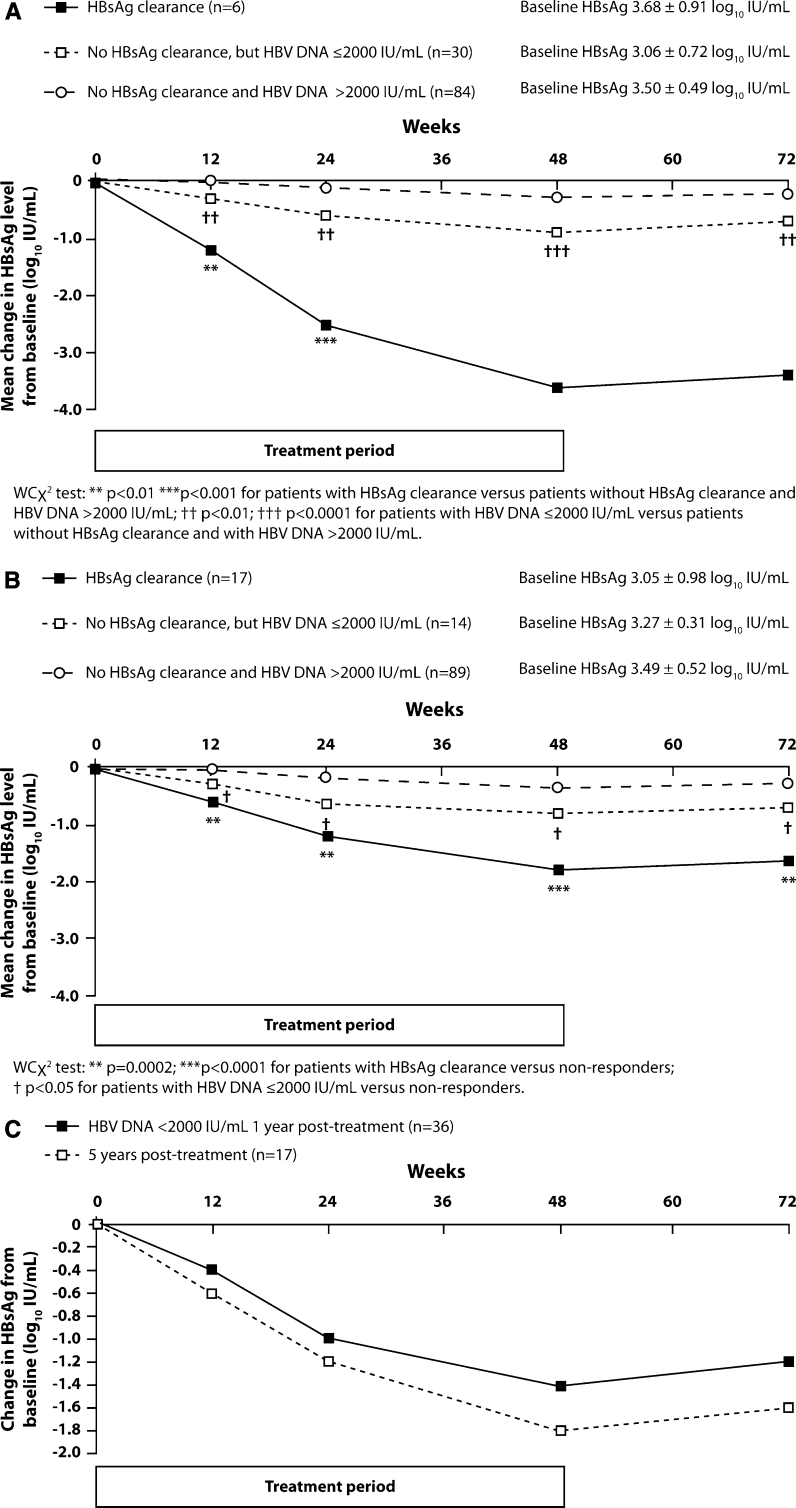
On-treatment HBsAg decline according to response. **a** 1 year post-treatment. **b** 5 years post-treatment. **c** 1 year (HBV DNA ≤2,000 IU/mL) and 5 years (HBsAg clearance) post-treatment

The patients were divided into three mutually exclusive response categories: (1) patients with HBsAg clearance, (2) patients without HBsAg clearance but with HBV DNA ≤2,000 IU/mL, and (3) patients without HBsAg clearance and with HBV DNA >2,000 IU/mL post-treatment. The three groups were determined based on response at year 1 (Fig. 1a) and at year 5 (Fig. 1b). HBsAg decline during treatment (48 weeks) and the initial follow-up period (24 weeks) was significantly more pronounced in patients with HBsAg clearance at either 1 or 5 years post-treatment when compared with patients not achieving HBsAg clearance or HBV DNA suppression. In patients with HBV DNA <2,000 IU/mL at 1 year post-treatment, the pattern of HBsAg decline was similar to that in patients with HBsAg clearance at 5 years post-treatment, although the overall decline was the greatest in patients with HBsAg clearance at 5 years post-treatment (Fig. 1c).

### HBsAg and HBV DNA decline in responders, relapsers, and non-responders

**Fig. 2:**
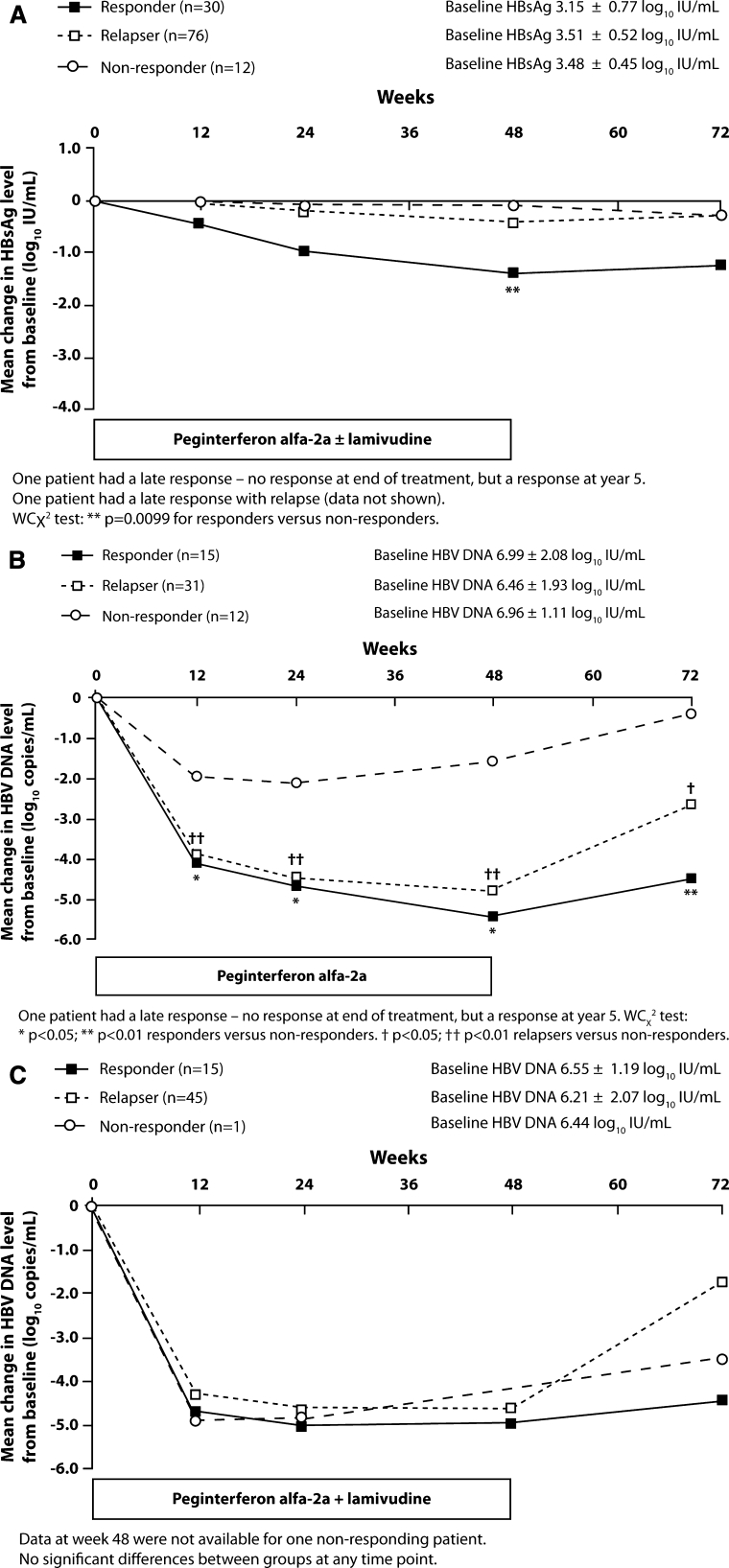
**a** On-treatment HBsAg decline in responders, relapsers, and non-responders. **b** On-treatment HBV DNA decline in responders, relapsers, and non-responders treated with peginterferon alfa-2a. **c** On-treatment HBV DNA decline in responders, relapsers, and non-responders treated with peginterferon alfa-2a + lamivudine. Responder: HBV DNA ≤2,000 IU/mL at end of treatment and year 5; relapser: HBV DNA <2,000 IU/mL at end of treatment; HBV DNA >2,000 IU/mL at year 5; non-responder: HBV DNA >2,000 IU/mL at end of treatment and year 5

The decline in HBsAg level in peginterferon alfa-2a ± lamivudine-treated patients with HBV DNA ≤2,000 IU/mL at the end of treatment and 5 years post-treatment (virologic responders, *n* = 31) was compared with that in patients with a response at end of treatment that was not sustained until year 5 (relapsers, *n* = 76) and in patients with HBV DNA >2,000 IU/mL at the end of treatment and 5 years post-treatment (non-responders; *n* = 13) (Fig. 2a). HBsAg clearance was more pronounced in responders than in relapsers and non-responders. Of the virologic responders, 17 (55%) achieved HBsAg clearance at 5 years post-treatment; however, none of the relapsing or non-responding patients achieved this endpoint.

In peginterferon alfa-2a-treated patients, on-treatment HBV DNA decline was similar in responders (*n* = 15) and relapsers (*n* = 31). The decline was significantly higher in responders and relapsers compared with non-responders (Fig. 2b). There was only one non-responder in the peginterferon alfa-2a + lamivudine-treated group, thus, making comparisons between groups difficult (Fig. 2c). On-treatment HBV DNA levels did not distinguish between responders and relapsers in either peginterferon alfa-2a treatment group.

### Association between on-treatment HBsAg level and response at 1 and 5 years post-treatment

Receiver operating characteristic analysis identified a ≥10% log_10_ HBsAg decline from baseline that was significantly associated with post-treatment response. The association between on-treatment HBsAg log_10_ decline and HBV DNA ≤2,000 IU/mL at 1 or 5 years post-treatment was investigated, using 10% log_10_ decline from baseline as a cut-off at weeks 12 and 24. More patients achieved ≥10% HBsAg log_10_ decline from baseline at week 24 (56%) than at week 12 (44%).

**Fig. 3:**
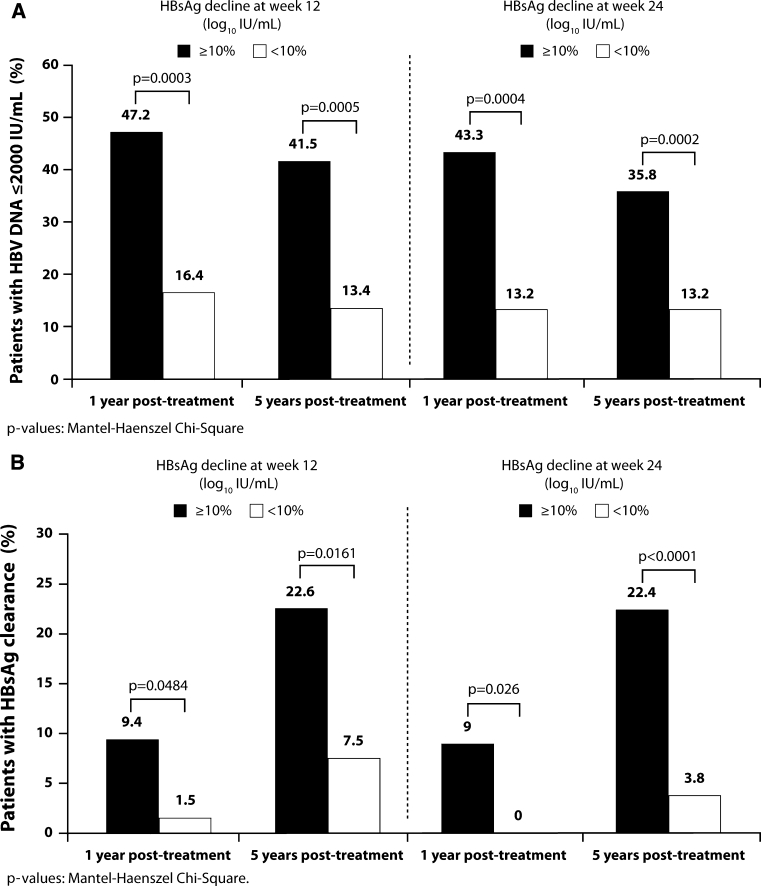
Response rates at 1 and 5 years post-treatment according to HBsAg decline at week 12 or week 24 of treatment. **a** Response defined as HBV DNA ≤2,000 IU/mL. **b** Response defined as HBsAg clearance

Patients with a ≥10% log_10_ HBsAg decline from baseline achieved significantly higher rates of HBV DNA ≤2,000 IU/mL at both year 1 and year 5 post-treatment than patients with a <10% log_10_ decline from baseline (Fig. 3a). Applying the identified cut-off level at weeks 12 and 24 provided PPVs of 47 and 43%, respectively, and NPVs of 84 and 87%, respectively, at year 1. At year 5, PPVs were 42 and 36%, respectively, and NPVs were 87% at both weeks 12 and 24.

Rates of HBsAg clearance were also significantly higher in patients with a ≥10% log_10_ decline in HBsAg from baseline at week 12 (*p* = 0.0484 for response at 1 year post-treatment and *p* = 0.0184 for response at 5 years post-treatment) and week 24 (*p* = 0.026 for response at 1 year post-treatment and *p* = 0.0038 for response at 5 years post-treatment) than in patients with a <10% log_10_ decline (Fig. 3b). The PPV and NPV for HBsAg clearance at 5 years post-treatment, based on ≥10% log_10_ decline in HBsAg from baseline at week 12, were 23 and 93%, respectively. At week 24, the PPV and NPV were 22 and 96%, respectively.

High rates of HBsAg clearance at 5 years post-treatment were achieved by patients with a ≥10% decline in HBsAg from baseline at week 12 and HBV DNA <2,000 IU/mL at 1 year post-treatment (40.0%, 10/25). Similarly, 44.8% (13/29) of patients with a ≥10% decline in HBsAg from baseline at week 24 and HBV DNA <2,000 IU/mL at 1 year post-treatment achieved HBsAg clearance at 5 years post-treatment.

## Discussion

The pivotal trial of peginterferon alfa-2a in HBeAg-negative CHB has provided a wealth of information about the long-term post-treatment effects of a finite course of treatment. A previous analysis of this study demonstrated that rates of HBsAg clearance increased after completion of treatment, with 9% of patients treated with peginterferon alfa-2a ± lamivudine achieving HBsAg clearance 3 years after treatment [7]. In the protocol-defined analysis described, rates of HBsAg clearance were shown to further increase during longer-term follow-up, with 12% of patients achieving HBsAg clearance at 5 years post-treatment. HBsAg clearance is the ultimate treatment goal of patients with HBeAg-negative CHB as it is associated with improved outcomes [3–5]. Although HBsAg clearance has been observed during treatment of HBeAg-positive CHB with some of the newer nucleos(t)ide analogs [18, 19], the rates of HBsAg clearance in HBeAg-negative patients during nucleos(t)ide analog therapy are negligible [19]. In contrast, the current study clearly demonstrates that rates of HBsAg clearance after peginterferon alfa-2a therapy are substantial and durable. Patients achieving HBV DNA suppression at 1 year post-treatment achieved high rates of HBsAg clearance at 5 years post-treatment. As 28% of patients with sustained immune control (HBV DNA ≤2,000 IU/mL at 1 year post-treatment) achieved HBsAg clearance at 5 years post-treatment, this endpoint appears to be a valuable early indicator of long-term response.

The analyses conducted in patients enrolled in this international, multicenter, randomized trial have considerably increased the knowledge of the potential value of peginterferon alfa-2a therapy in patients with HBeAg-negative CHB. As a result of the heterogeneous population enrolled, this study—the largest and the most extensive of peginterferon alfa-2a in HBeAg-negative CHB to date—closely reflects the global clinical situation. The long-term benefits achieved following a finite course of peginterferon alfa-2a in such a population are clearly encouraging. Early identification of patients who could benefit from this treatment approach would be valuable, as it would allow clinicians to motivate patients likely to achieve a long-term response to complete therapy while also identifying those patients for whom an alternative treatment regimen may be necessary.

Recently, two groups showed that HBsAg levels vary considerably during the natural history of CHB and reflect the disease phase [20, 21], and previous results demonstrated that on-treatment HBsAg kinetics and HBsAg levels at the end of treatment are associated with sustained response to peginterferon alfa-2a [14, 17]. The observation that lower levels of HBsAg are associated with greater immune control has resulted in considerable interest in HBsAg kinetics during peginterferon alfa-2a therapy.

A difference in HBsAg decline between responders and non-responders, as occurred in this study, was described initially by Brunetto et al*.* [17]. In the current analysis, differences in HBsAg decline patterns were also observed in patients achieving a long-term response to treatment compared with those relapsing after experiencing an on-treatment response. This confirms data from Moucari et al. [14] that showed the association between HBsAg decline and short-term post-treatment response. As variations in HBsAg kinetics between responders, non-responders, and relapsers to peginterferon alfa-2a therapy have been established in both short-term and long-term follow-up, research has focused on whether clinicians can use the knowledge of HBsAg kinetics to make disease-management decisions.

In the current study, receiver operating characteristic analysis identified a decline in HBsAg levels at weeks 12 and 24, which was associated with high rates of post-treatment response. Patients with a ≥10% log_10_ IU/mL decline in HBsAg from baseline achieved significantly higher rates of HBV DNA ≤2,000 IU/mL and HBsAg clearance up to 5 years post-treatment than patients not achieving this level of decline. It is worth noting that 40–45% of patients with this level of on-treatment HBsAg decline and sustained immune control at 1 year post-treatment achieved HBsAg clearance at 5 years post-treatment. Although PPVs for long-term sustained immune control were slightly lower at week 24 than at week 12, the high rates of HBsAg clearance achieved by patients with a ≥10% decline during treatment and sustained immune control 1 year post-treatment suggest that on-treatment quantification at either time-point provides clinically important information.

The target was to achieve NPV ≥95%, as this would identify patients unlikely to achieve a sustained response and allow physicians to consider stopping treatment. However, as the NPVs generated by this analysis did not reach the target level, using this as a stopping rule would mean that 13–16% of potential responders would have their treatment stopped prematurely. Some smaller studies have identified on-treatment HBsAg cut-off levels that generate higher NPVs. For example, Moucari et al. [14] showed that an HBsAg decline of 0.5 log_10_ IU/mL (68% decline from baseline) at week 12 generated an NPV of 90% and an HBsAg decline of 1.0 log_10_ IU/mL (90% decline from baseline) at week 24 gave an NPV of 97%. However, these levels of decline did not produce similar results in the current analysis (data not shown). The lack of consistent findings may be explained by differences in the study design, response parameters, and study populations between the two studies. For example, Marcellin et al. [7] defined post-treatment response as undetectable HBV DNA at 6 months post-treatment, rather than the HBV DNA <2,000 IU/mL and HBsAg clearance at 1 year and 5 years post-treatment employed in the current analysis. Given that relapse is known to occur between 6 months and 1 year post-treatment [7], 1 year may be a more appropriate time-point for assessment of sustained response. In addition, the differences in cut-off levels identified in these two populations may be explained, at least in part, by HBV genotype, which is known to affect post-treatment response [14, 17, 22, 23]. In the current analysis, the patients were infected predominantly with HBV genotype C (46%) and D (22%) with very few infected with HBV genotype A (10%); while in the Moucari et al. [14] analysis, 27% of patients were infected with HBV genotype A. The influence of HBV genotype on HBsAg kinetics in samples from patients who were also included in the current analysis was described initially by Brunetto et al. [17] who showed that patients infected with all the major genotypes achieve some level of HBsAg decline, but this was most pronounced in patients infected with genotypes A and B. Genotype is, therefore, likely to be a major influencing factor on decline of HBsAg levels in HBeAg-negative subjects. Further analyses are needed to elucidate the influence of genotype on HBsAg kinetics and to determine how knowledge of infecting genotype combined with HBsAg quantification can be used to predict response to peginterferon alfa-2a.

While most investigations have examined on-treatment prediction of response, there is also potential value in identifying at baseline those patients likely to respond to peginterferon alfa-2a. In the current analysis, patients with HBsAg ≤5,000 IU/mL at baseline achieved the highest rates of response post-treatment, but the PPVs (approximately 30%) and NPVs (approximately 80%) calculated were lower than those relating to on-treatment HBsAg quantification. Consequently, on-treatment HBsAg quantification appears to be a more appropriate predictor than quantification of HBsAg at baseline.

Unlike on-treatment HBsAg quantification, measurement of HBV DNA levels during treatment does not differentiate between treatment responders and relapsers [14]. This observation was based on response at 6 months post-treatment but was also seen in the current analysis, where a significant difference in HBV DNA decline between patients with a response at 5 years post-treatment and relapsers could not be demonstrated. In addition, there were differences in HBV DNA decline between peginterferon alfa-2a-treated patients and patients receiving combination therapy with lamivudine. Wherever there was a difference in HBV DNA decline between responders/relapsers and non-responders in monotherapy-treated patients, this was not present in the combination therapy group. Consequently, HBV DNA quantification may not be as valuable for identifying long-term responders to therapy as HBsAg quantification.

A recent analysis of another study of peginterferon alfa-2a in HBeAg-negative patients investigated the potential of combining HBsAg and HBV DNA response during treatment to improve NPVs [24]. The NPV of 100% was reported in patients who did not achieve an HBsAg decline or an HBV DNA decline >2 log_10_ copies/mL at week 12 of treatment. The importance of infecting genotype on HBsAg kinetics was discussed earlier, and as most patients in this analysis were infected with HBV genotype D, further analysis is required to determine whether this rule can be used in patients infected with other genotypes.

It is interesting to speculate how HBsAg quantification could be used in clinical practice to help individualize peginterferon alfa-2a therapy. Preliminary data from a study of extended peginterferon alfa-2a therapy in HBeAg-negative patients have shown that extension therapy improves sustained response rates as a result of a reduction in relapse [25]. An important future consideration will be whether patients likely to benefit from extended therapy can be identified early during the initial phase of treatment. Brunetto et al. [26] have studied in-depth HBsAg kinetics in the HBeAg-negative patients included in the Phase 3 peginterferon alfa-2a study and showed that response rate is linked to decline pattern. Although patients with a continuous HBsAg decline from baseline (≥10% decline from baseline to week 24 and ≥10% from weeks 24 to 48) achieved the highest rates of response, patients with a late decline (≥10% decline from baseline after week 24) also achieved high rates of response when compared with patients with a <10% HBsAg decline during the entire 48-week treatment period. It is possible that patients with a late HBsAg decline will benefit from an extended period of peginterferon alfa-2a therapy; however, this needs to be studied in prospective clinical trials, which also consider in more detail the role of infecting genotype.

The current analysis has limitations. Only a proportion of patients included in the initial or follow-up studies had HBsAg levels determined during treatment and 6 months post-treatment. In addition, only patients with HBsAg data available at all on-treatment and post-treatment time-points were included and, consequently, there is the potential for selection bias. However, baseline characteristics and response rates in the 230 patients in the long-term analysis were similar to those achieved by the 120 patients in the current analysis, and the statistical methods used were conservative as missing samples were taken as non-responders. Wherever LOCF methodology was used, a secondary parameter was included (HBV DNA <71 IU/mL) to reduce the chance of false-positive data.

In conclusion, a finite course of peginterferon alfa-2a resulted in increasing rates of HBsAg clearance up to 5 years post-treatment in HBeAg-negative patients. Sustained immune control at 1 year post-treatment was an early indicator of subsequent HBsAg clearance. Analysis of data from this large, long-term study has shown that HBsAg quantification may be an appropriate on-treatment tool for monitoring response to peginterferon alfa-2a in HBeAg-negative patients, thereby confirming observations in small-scale studies. Further prospective studies are required before clear clinical guidance on use of HBsAg monitoring can be provided for physicians. In the future, increased understanding of the kinetics of HBsAg decline in HBeAg-negative patients treated with peginterferon alfa-2a may help physicians make individualized treatment decisions that should ultimately increase the rate of response in patients with CHB.
